# Impact of leukoaraiosis severity on the association of outcomes of mechanical thrombectomy for acute ischemic stroke: a systematic review and a meta-analysis

**DOI:** 10.1007/s00415-020-10167-0

**Published:** 2020-08-28

**Authors:** Longwen Huo, Penghui Chen, Zhongxiu Wang, Xiandong Li, Jie Zhou, Chao Wang, Dajiang Xing, Shouchun Wang

**Affiliations:** 1 Department of Neurology and Neuroscience Center, The First Hospital of Jilin University, Changchun, China; 2 Department of Cardiology, Hunan Provincial People’s Hospital, The First Affiliated Hospital of Hunan Normal University, Changsha, China

**Keywords:** Leukoaraiosis, Thrombectomy, Stroke, Large vessel occlusion

## Abstract

**Background:**

Leukoaraiosis (LA) severity is associated with poor outcome after mechanical thrombectomy (MT) for acute ischemic stroke (AIS) caused by large vessel occlusion. This meta-analysis aimed to assess the association of LA severity with AIS-related risk factors and outcomes of MT.

**Methods:**

PubMed, Web of Science, EMBASE, and Cochrane Collaboration Database was searched for studies on MT for AIS with LA. We conducted a random-effects meta-analysis for the prevalence of stroke risk factors and the MT outcome in the absent to moderate LA and severe LA groups.

**Results:**

We included seven cohort studies involving 1294 participants (1019 with absent to moderate LA and 275 with severe LA). The absent to moderate LA group had a significantly lower prevalence of coronary artery disease (odds ratio [OR] 0.43; 95% CI 0.29–0.66), atrial fibrillation (OR, 0.26; 95% CI 0.17–0.38), hypertension (OR, 0.39; 95% CI 0.24–0.61), and ischemic stroke (OR, 0.27; 95% CI 0.15–0.50) than the severe LA group. There were no significant between-group differences in symptom onset to recanalization time (364.4 versus 356.2 min, mean difference 19.4; 95% CI − 28.3 to 67.2), final recanalization rate (modified thrombolysis in cerebral infarction score of 2b/3; OR, 0.87; 95% CI 0.55–1.38), and symptomatic intracranial hemorrhage (OR, 0.62; 95% CI 0.34–1.11). The absent to moderate LA group had a higher good functional outcome (modified Rankin Scale score of 0–2 at 90 days; OR, 4.55; 95% CI 3.20–6.47) and a lower mortality rate (179/1019 vs 108/275; OR, 0.28; 95% CI 0.20–0.39).

**Conclusion:**

There are unique differences in the characteristics of risk factors and clinical outcomes of ischemic stroke across patients with LA of different severity. Patients with severe LA are more likely to be associated with risk factors for cerebrovascular disease and have a poor post-MT outcome.

**Electronic supplementary material:**

The online version of this article (10.1007/s00415-020-10167-0) contains supplementary material, which is available to authorized users.

## Introduction

Stroke is among the main mortality and disability causes with its treatment crucially relying on early revascularization and rescue ischemic penumbra. Five randomized controlled trials reported that [1] mechanical thrombectomy (MT) could improve the functional outcome after acute ischemic stroke (AIS) caused by anterior circulation large vessel occlusion (LVO). This is the highest available evidence level of a treatment technique available according to the latest guidelines from American Heart Association/American Stroke Association (AHA/ASA) [2]. Moreover, indications for MT have been recently expanded to benefit more patients, including a longer time window [3–5], lower National Institutes of Health Stroke Scale (NIHSS) score [6], larger infarct cores [7], and elderly patients [8]. However, the reported rate of good post-MT functional outcome (modified Rankin Scale [mRS] score of 0–2 at 90 days) was < 50% [1]. Reported futile recanalization causes include old age, moderate and severe stroke, surgical- and instrument-related complications, delayed opening of blood vessels, distal vascular embolisms, and reocclusion [9–11]. Therefore, it is important to recognize poor outcome factors and select the most suitable patients for MT to improve the proportion of good outcomes.

Leukoaraiosis (LA) is a common neuroimaging feature of cerebral small vessel disease. On MRI, it presents as a more or less symmetrical fusion region of white matter in the left and right cerebral hemispheres, which shows hyperintensity in T2-weighted and fluid-attenuated inversion recovery images [12]. Recently, there have been increasing studies on the relationship between AIS and leukoaraiosis. Based on the association of white matter hyperintensity volumes with the 3-month functional post-stroke outcome, patients with severe LA were found to have a poor post-stroke outcome. Moreover, higher white matter hyperintensity is associated with poor improvement of the mRS score [13]. In addition, a previous systematic review and meta-analysis showed a close association of LA presence and severity with an increased risk of cerebral hemorrhage and poor functional outcome after thrombolysis for AIS [14]. Regarding endovascular therapy, there was a positive correlation of white matter hyperintensity (WMH) volumes and the risk of unfavorable functional outcome. However, there was no correlation of WMH severity with the rate of symptomatic intracerebral hemorrhage (sICH) [15]. However, studies have reported that post-MT clinical outcome is not associated with white matter burden in patients with AIS caused by LVO [16, 17]. Thus, the relationship between LA severity and the clinical outcome of MT for AIS remains unclear. This systematic review and meta-analysis aimed to improve the understanding of the clinical characteristics and thrombectomy outcome in patients with different LA severities.

## Methods

### Searching strategy

This systematic review and meta-analysis were based on PRISMA reports [18]. We systematically retrieved English articles from PubMed, EMBASE, Web of Science, and Cochrane library up to May 23, 2020, using the following terms as keywords or MeSH terms: white matter*, leukoaraiosis, Leukoencephalopathies, Leukoencephalopathy, Stroke, Brain Infarction, Strokes, Cerebrovascular Accident, Cerebrovascular Accidents, Cerebrovascular Apoplexy, Cerebral Infarction, thrombectomy, Thrombectomie, Percutaneous Aspiration Thrombectomy, Aspiration Thrombectomy, Aspiration Thrombectomies, endovascular therapy, and endovascular treatment. Details regarding the search strategies are provided in the online supplementary figure 1. Further, we conducted a manual search for the references to include published articles and pertinent reviews and ensure more comprehensive literature retrieval.

### Inclusion and exclusion criteria

The inclusion criteria for studies were: (1) to evaluate observational studies in patients with AIS; (2) all patients received endovascular therapy, excluding comparisons between thrombolytic and conservative drug therapy; (3) sample size of > 50 patients; (4) different LA severities classified according to the LA scales, including Fazekas scale [19] and Van Swieten scale [20]; (5) comparison of the clinical features and risk factors of different LA severities; and (6) reporting thrombectomy and clinical outcome of different LA severities. We excluded the following studies: (1) conference abstracts, reviews, letters, editorial, case reports, or animal studies; (2) non-comparative study or inappropriate groupings; (3) no assessment of relevant outcomes.

### Data extraction

Two experienced investigators (LH, PC) independently extracted baseline data and main outcomes with any disagreements being resolved by a third investigator (ZW). We collected the following baseline data and main outcomes: demographic profiles (total number of patients, age, and sex), baseline systolic blood pressure (SBP), baseline NIHSS score, pre-existing risk factors (coronary artery disease, atrial fibrillation, hypertension, diabetes mellitus, hyperlipidemia, and ischemic stroke); stroke mechanism (large artery atherosclerosis, cardioembolic, undetermined, or others); intravenous thrombolysis; and main outcomes (symptom onset to recanalization time, successful recanalization [mTICI 2b/3], mRS score of 0–2 at 90 days, symptomatic intracranial hemorrhage [sICH], and mortality at 90 days). Moreover, we defined a van Swieten and Fazekas scale scores of 0–2 as absent to moderate LA while a van Swieten scale 3–4 and Fazekas scale 3–6 were defined as severe LA.

### Quality assessment

For quality evaluation of non-randomized studies, we used the Newcastle–Ottawa Scale to evaluate the included studies. Three aspects were used to evaluate the included studies: election of cohorts, comparability of cohorts and assessment of outcome [21]. To assess the risk of bias, the two investigators (LH, PC) conducted an independent assessment with any resulting disagreements being settled by a third investigator (XL).

### Statistical analysis

We analyzed the following outcomes: demographic profiles, baseline SBP, baseline NIHSS score, pre-existing risk factors, stroke mechanism, tangible thrombolysis, and main clinical outcomes. Outcome meta-analysis was conducted using the random-effect model. We calculated the odds ratio (OR) of binary variables or the mean difference (MD) of continuous variables with the 95% confidence intervals (CIs). We evaluated inter-study heterogeneity using *I*^2^ statistics where significant heterogeneity was defined at *P* < 0.10, *I*^2^ > 50%. In the case of significant heterogeneity, we conducted sensitivity and subgroup analysis to analyze the heterogeneity sources of main clinical outcomes. We performed a subgroup analysis of the LA scale scores. In the sensitivity analysis, the effect of one study on the total pooled effect size. Publication bias was assessed using funnel plots and Egger's test (significance threshold set at *P* < 0.10). In case the single analysis included > 6 studies, we conducted Egger's test.

Meta-analysis and statistical analysis were conducted using Stata 16.0 (StataCorp, College Station, TX, USA).

## Results

### Search results

**Fig. 1:**
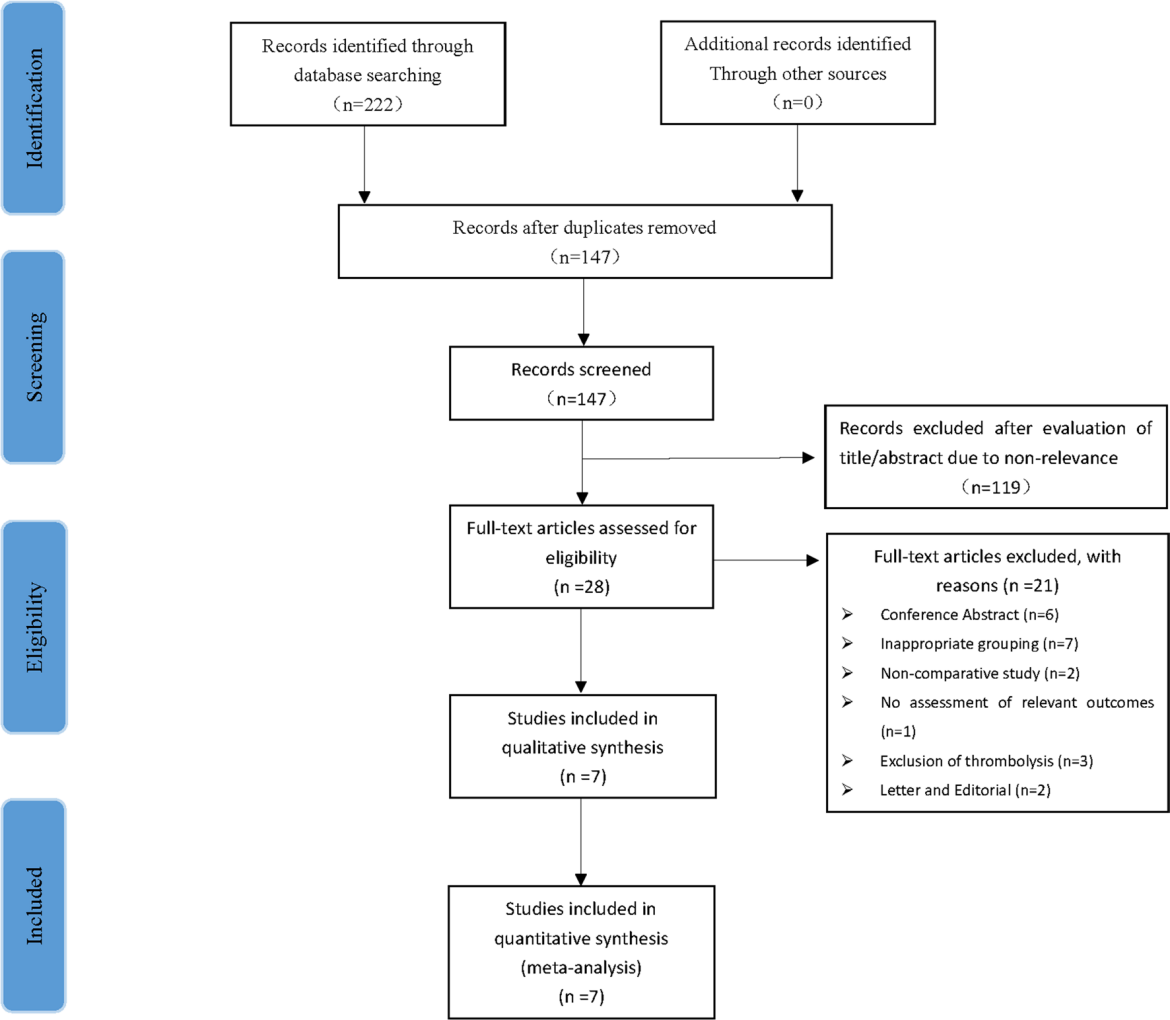
Flowchart study of selection process

Using a comprehensive search strategy, we retrieved 222 original articles. Among these articles, 39, 80, 101, and 2 were retrieved from PubMed, Web of Science, EMBASE, and Cochrane Collaboration Database, respectively. Among them, Seventy-five articles were repetitive with the remaining 147 articles being screened by the title, abstract, and full text. Finally, 7 original studies [22–28] were included, which included a prospective cohort study [28] and six retrospective cohort studies [22–27]. Figure 1 presents a flow chart of the specific study selection process.

### Characteristics of the eligible studies

The included 7 cohort studies enrolled 1294 patients with AIS, including 1019 and 275 patients with absent to moderate LA and severe LA, respectively. Two and five studies were performed in China [24, 25] and The United States, respectively [22, 23, 26–28]. In addition, two studies [22, 25] used the Fazekas Scale to classify LA severity while five studies [23, 24, 26–28] used the van Swieten Scale. We used the Newcastle–Ottawa Scale to assess the risk of bias in cohort studies, including selection, comparability, and outcome biases, which indicated a low risk of bias. Table 1 shows the study characteristics of the included articles.

**Table 1:** Patient population and study design of included articles**

Author	Year	Absent to moderate LA	Severe LA	Population	Study Design	LA Scale	Risk of Bias (NOS)
Shi et al.	2012	79	26	United States	Retrospective, single-center	Fazekas Scale	7
Zhang et al.	2014	100	29	United States	Retrospective, single-center	van Swieten Scale	8
Guo et al.	2019	213	38	China	Retrospective, multicenter	van Swieten Scale	8
Liu et al.	2019	43	54	China	Retrospective, single-center	Fazekas Scale	8
Albo et al.	2020	148	33	United States	Retrospective, single-center	van Swieten Scale	8
Mikati et al.	2020	122	22	United States	Retrospective, single-center	van Swieten Scale	8
Mistry et al.	2020	315	74	United States	Prospective, multicenter	van Swieten Scale	8

### Patient characteristics and risk factors

According to demographic profiles, there were significant between-group differences in the age (64.6 vs. 77.3; mean difference, − 13.57; 95% CI − 15.71 to − 11.43) and proportion of male patients (57.3% vs. 48.4%; OR, 1.55; 95% CI 1.09–2.19).

Regarding stroke risk factors, the absent to moderate LA group had a significantly lower prevalence of coronary artery disease (22.6% vs. 41.0%; OR, 0.43; 95% CI 0.29–0.66), atrial fibrillation (36.9% vs. 66.8%; OR, 0.26; 95% CI 0.17–0.38), hypertension (66.3% vs. 83.3%; OR, 0.39; 95% CI 0.24–0.61), and ischemic stroke (11.8% vs. 29.6%; OR, 0.27; 95% CI 0.15–0.50) compared with the severe LA group. There were no significant between-group differences in the diabetes mellitus (21.5% vs. 26.0%; OR, 0.73; 95% CI 0.49–1.09), and hyperlipidemia (53.2% vs. 59.5%; OR, 0.79; 95% CI 0.49–1.28). Regarding stroke mechanism, there were no significant between-group differences in large artery atherosclerosis (28.3% vs. 20.5%; OR, 1.43; 95% CI 0.87–2.36); however, there were significant differences in cardioembolic (48.2% vs. 73.8%; OR, 0.28; 95% CI 0.10–0.77). Regarding baseline SBP and baseline NHISS score, there were significant between-group differences. Table 2 shows the outcome of all the characteristics.

**Table 2:** Clinical features and risk factors of absent to moderate LA versus severe LA

	Absent to moderate LA (%)	Severe LA (%)	OR (95% CI)	*P* value	*I*^2^ (*P* value)
Male*	57.3	48.4	1.546 (1.089–2.194)	0.015	26.5% (0.236)
Coronary artery disease*	22.6	41.0	0.434 (0.286–0.659)	< 0.001	0.0% (0.474)
Atrial fibrillation*	36.9	66.8	0.258 (0.174–0.383)	< 0.001	17.4% (0.301)
Hypertension*	66.3	83.3	0.386 (0.244–0.611)	< 0.001	32.5% (0.180)
Diabetes mellitus	21.5	26.0	0.729 (0.486–1.093)	0.126	20.8% (0.277)
Hyperlipidemia	53.2	59.5	0.788 (0.486–1.278)	0.334	0.0% (0.898)
Ischemic stroke*	11.8	29.6	0.272 (0.148–0.501)	< 0.001	57.6% (0.051)
Large artery atherosclerosis	28.3	20.5	1.432 (0.869–2.358)	0.159	0.0% (0.503)
Cardioembolic*	48.2	73.8	0.282 (0.103–0.771)	0.014	77.2% (0.004)
Undetermined or others*	23.3	6.6	3.700 (1.388–9.865)	0.009	38.4% (0.182)
IV thrombolysis	47.3	47.1	1.019 (0.624–1.665)	0.939	60.6% (0.018)
Mean and mean difference (95% CI)					
Age(years)*	64.6	77.3	− 13.573 (− 15.713 to − 11.433)	< 0.001	48.8% (0.069)
Baseline SBP*	145.3	152	− 7.878 (− 11.635 to -4.121 )	< 0.001	0.0% (0.478)
Baseline NIHSS score*	15.9	17.5	− 2.342 (− 3.480 to − 1.205 )	< 0.001	32.6% (0.179)

*IV* intravenous, *SBP* systolic pressure, *NIHSS* National Institutes of Health Stroke Severity Scale, *OR* odds ratio

*P < 0.05

### Clinical outcomes

**Fig. 2:**
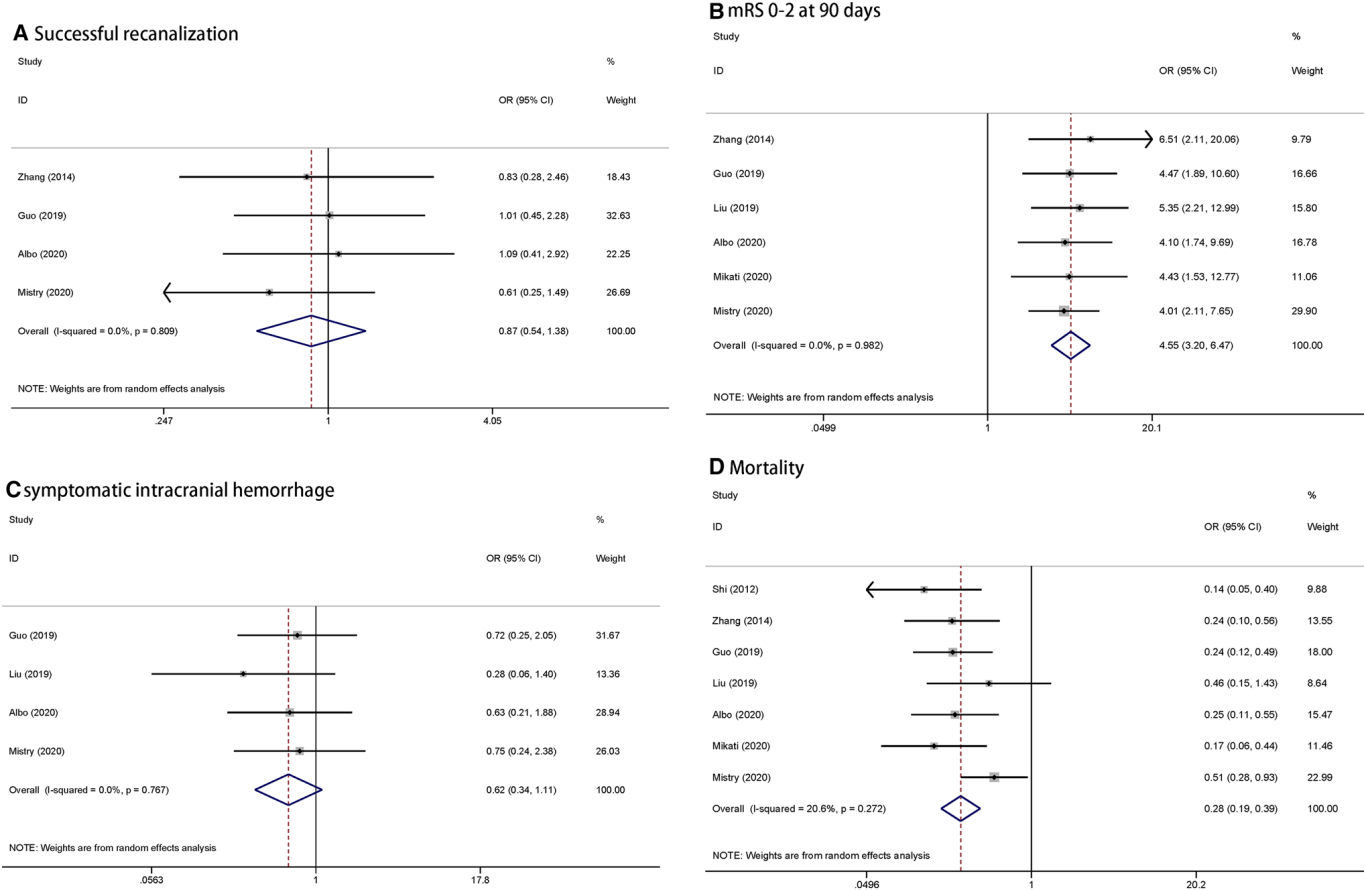
**a** Successful recanalization, **b** mRS 0–2 at 90 days, **c** symptomatic intracranial hemorrhage, **d** mortality

There were no significant between-group differences in the symptom onset to recanalization time (364.4 vs. 356.2 min; mean difference, 19.42; 95% CI − 28.35 to 67.18), the final recanalization rate (mTICI 2b/3; 82.6% vs. 85.1%; OR, 0.87; 95% CI 0.55–1.38), and sICH (7.1% vs. 11.1%; OR, 0.62; 95% CI 0.34–1.11). However, there was a significant between-group difference in functional independence (mRS 0–2 at day 90; 51.9% vs. 19.9%; OR, 4.55; 95% CI 3.20–6.47) and 90-day mortality rates (17.6% vs. 39.3%; OR, 0.28; 95% CI 0.19–0.39). These results are shown in Fig. 2 and Table 3.

**Table 3:** Summary of meta-analysis outcomes of thrombectomy in absent to moderate LA versus severe LA

	Absent to moderate LA (%)	Severe LA (%)	OR (95% CI)	*P* value	I^2^ (P Value)
mTICI 2b/3	82.6	85.1	0.866 (0.545–1.378)	0.545	0.0% (0.809)
mRS score of 0–2 at 90 d*	51.9	19.9	4.549 (3.198–6.471)	< 0.001	0.0% (0.982)
sICH	7.1	11.1	0.619 (0.344–1.113)	0.109	0.0% (0.767)
Mortality at 90 d*	17.6	39.3	0.275 (0.193–0.393)	< 0.001	20.6% (0.272)
Mean and mean difference (95% CI)					
OTR	364.4	356.2	19.417 (− 28.345 to 67.179)	0.426	74.4% (0.004)

*mTICI* modified Thrombolysis in Cerebral Infarction, *mRS* modified Rankin Scale, *sICH* symptomatic intracranial hemorrhage, *OTR* symptoms onset to recanalization time

*P < 0.05

### Study heterogeneity

There was low study heterogeneity (*I*^2^ < 50%) as follows: age (*I*^2^ = 48.8%), proportion of male patients (*I*^2^ = 26.5%), baseline SBP (*I*^2^ = 0%), baseline NIHSS score (*I*^2^ = 32.6%), coronary artery disease (*I*^2^ = 0%), atrial fibrillation (*I*^2^ = 17.4%), hypertension (*I*^2^ = 32.5%), diabetes mellitus (*I*^2^ = 20.8%), hyperlipidemia (*I*^2^ = 0%), large artery atherosclerosis (*I*^2^ = 0%), undetermined or others (*I*^2^ = 38.4%), final rate of mTICI 2b/3 (*I*^2^ = 0%), mRS score of 0–2 at 90 days (*I*^2^ = 0%), symptomatic intracranial hemorrhage (*I*^2^ = 0%), and mortality at 90 days (*I*^2^ = 20.6%). These results showed moderate substantial heterogeneity (*I*^2^ > 50%): ischemic stroke (*I*^2^ = 57.6%), cardioembolic (*I*^2^ = 77.2%), intravenous thrombolysis (*I*^2^ = 60.6%), and symptom onset to recanalization time (*I*^2^ = 74.4%).

### Sensitivity analysis and publication bias

Given the small number of included studies and low heterogeneity of clinical outcomes, we did not conduct a subgroup analysis of all studies. Regarding outcome indications for > 6 studies, the sensitivity analysis shows that the results were statistically stable and reliable (online supplementary figure 2). Further, we used the funnel plot to qualitatively analyze the publication bias and observed possibly symmetrical distribution of these studies (online supplementary figure 3). Further, we used the Egger's test quantitative analysis of publication bias and concluded that neither mortality at 90 days (*P* = 0.20) nor functional independence (*P* = 0.11) had obvious publication bias.

## Discussion

To our knowledge, this is the first systematic review and meta-analysis to investigate the effect of leukoaraiosis severity on the association of MT outcomes for AIS. Our findings suggest that patients with severe LA may be older, as well as have more relevant risk factors (e.g. coronary artery disease, atrial fibrillation, hypertension, ischemic stroke) and higher NIHSS scores. Therefore, post-MT patients may have worse functional independence and a higher mortality rate. However, the LA severity does not seem to affect the rate of successful recanalization and sICH.

There is a high LA prevalence in patients with AIS, which increases with age and is as high as 95% in people aged 60–90 years [29]. Furthermore, women tend to have greater white matter lesions than men [30], which is consistent with our findings. LA is currently considered an independent predictor of unfavorable outcome for AIS [24, 26, 31, 32]. Further, LA affects the AIS severity, including infarct volume, collateral circulation, NIHSS score, etc. Henninger et al. [33] retrospectively analyzed 117 patients with middle cerebral artery occlusion and graded LA severity using the van Swieten scale. They found an independent correlation of severe LA with infarct volume > 27 ml where the latter was an independent predictor of unfavorable 90-day outcome. Therefore, LA severity is associated with greater infarct volume and unfavorable functional outcome. Helenius et al. [34] assessed the effect of LA severity on infarct size and NIHSS in 312 patients with supratentorial ischemic stroke by analyzing NIHSS and the diffusion-weighted imaging-defined infarct volume. Multivariable linear regression models indicated an independent association of LA and infarct volume with a larger NIHSS deficit. Similarly, we observed a positive association of the NIHSS score with LA severity. However, further studies should assess this association. Mark et al. [35] assessed the relationship between LA and collateral circulation patients with stroke with anterior circulation great vessel occlusion by performing a retrospective analysis of 178 patients with anterior circulation great vessel occlusion who were classified according to collateral grade. The Fazekas scale assesses LA severity. We observed a negative correlation of the total Fazekas score with collateral circulation; moreover, severe LA was associated with poor collateral grade among patients with AIS. Further, LA affected the short- and long-term AIS outcomes. Guo et al. [36] performed a multi-center retrospective analysis of 273 patients with acute stroke after MT and assessed early neurological improvement and deterioration according to the classification on the Van Swieten. Multivariate analysis revealed a negative correlation of severe LA with early neurological improvement. Among patients with asymptomatic intracranial hemorrhage, severe LA is an independent predictor of early neurological deterioration. Furthermore, Georgakis et al. [37] conducted a meta-analysis of the LA effects and long-term outcome in ischemic stroke. The longest follow-up time in the included studies was 15 years. They reported a positive association of LA severity with post-ischemic stroke dementia, dysfunction, stroke recurrence, and mortality. Similarly, this meta-analysis found similar findings of long-term AIS outcome. However, some studies have suggested that no LA effect on the AIS outcome. Atchaneeyasakul et al. [17] conducted a retrospective analysis on 56 patients to assess the association between white matter hyperintensity volume and patients with AIS undergoing MT using new-generation devices. They concluded that white matter hyperintensity volume did not affect MT outcome, including good functional, successful recanalization, mortality, and any cerebral hemorrhage.

The exact pathologic mechanism underlying the association of LA severity with unfavorable outcomes in post-MT patients with AIS remains unclear. There have been reports of an association of LA severity with older, uncontrolled hypertension and hyperhomocysteinemia, which are risk factors for unfavorable outcome in AIS [38, 39]. Regarding LA-associated mechanisms, LA has been reported to be related with microcirculation disorder, which causes small vessel narrowing, elongation, and tortuosity; moreover, this could lead to reduced cerebral blood flow [12]. Therefore, for patients with AIS, LA severity could be negatively associated with the survival rate of ischemic penumbra, which impedes good tissue outcome [40]. Second, LA is associated with platelet activation and hypercoagulability [41, 42]. Reopening of the occluded vessel reopening does not completely restore the microcirculatory blood flow of ischemic tissues [43]. Moreover, capillary contraction and perivascular astrocyte swelling aggravate the hemodynamic disorder, which leads to futile recanalization. In addition, LA severity was found to be associated with poor collateral grade in post-MT patients with AIS [44]. This indicates that patients with LA may present with damaged cerebral collateral circulation; moreover, their brain tissue may have reduced resistance to ischemia, which leads to a larger infarct core. Finally, oxidative stress, damage to the blood–brain barrier, axon and oligodendrocyte loss, and other changes are closely associated with severe LA occurrence and development, which has various effects on stroke outcome [45].

This meta-analysis has several limitations. First, there was no uniform definition for LA severity among the included studies, which affects group disagreement. Second, most of the included studies were from North America with only two coming from Asia, which increased the risk of bias. Clinical characteristics and outcomes may differ for patients in other regions, including Europe. Third, our sample size was relatively small. Finally, the included studies lacked statistical indicators of adverse outcomes, including reocclusion, sICH, parenchymal hemorrhage, etc. There is a need for future multicenter prospective cohort studies with larger sample sizes and more comprehensive outcome indicators.

## Conclusion

In conclusion, for post-MT patients with AIS, preoperative CT or MRI examinations are required; however, but the LA severity is often overlooked. Patients with differences in LA severity exhibit unique risk factor characteristics. Patients with severe LA may have a lower 90-day functional outcome and higher mortality. However, it does not affect the reperfusion proportion. Thus, the presence and severity of LA should not be denied as a standard treatment for patients with AIS.

## Electronic supplementary material

Below is the link to the electronic supplementary material.

Supplementary file1 (TIFF 382kb)

Supplementary file2 (PNG 49kb)

Supplementary file3 (TIFF 5522kb)

## Data Availability

Not applicable.
