# Supplementary figures and images for: Impact of leukoaraiosis severity on the association of outcomes of mechanical thrombectomy for acute ischemic stroke: a systematic review and a meta-analysis
Source: J Neurol. 2020 Aug 28;268(11):4108–16. doi: 10.1007/s00415-020-10167-0 (PMC8505273; doi:10.1007/s00415-020-10167-0)

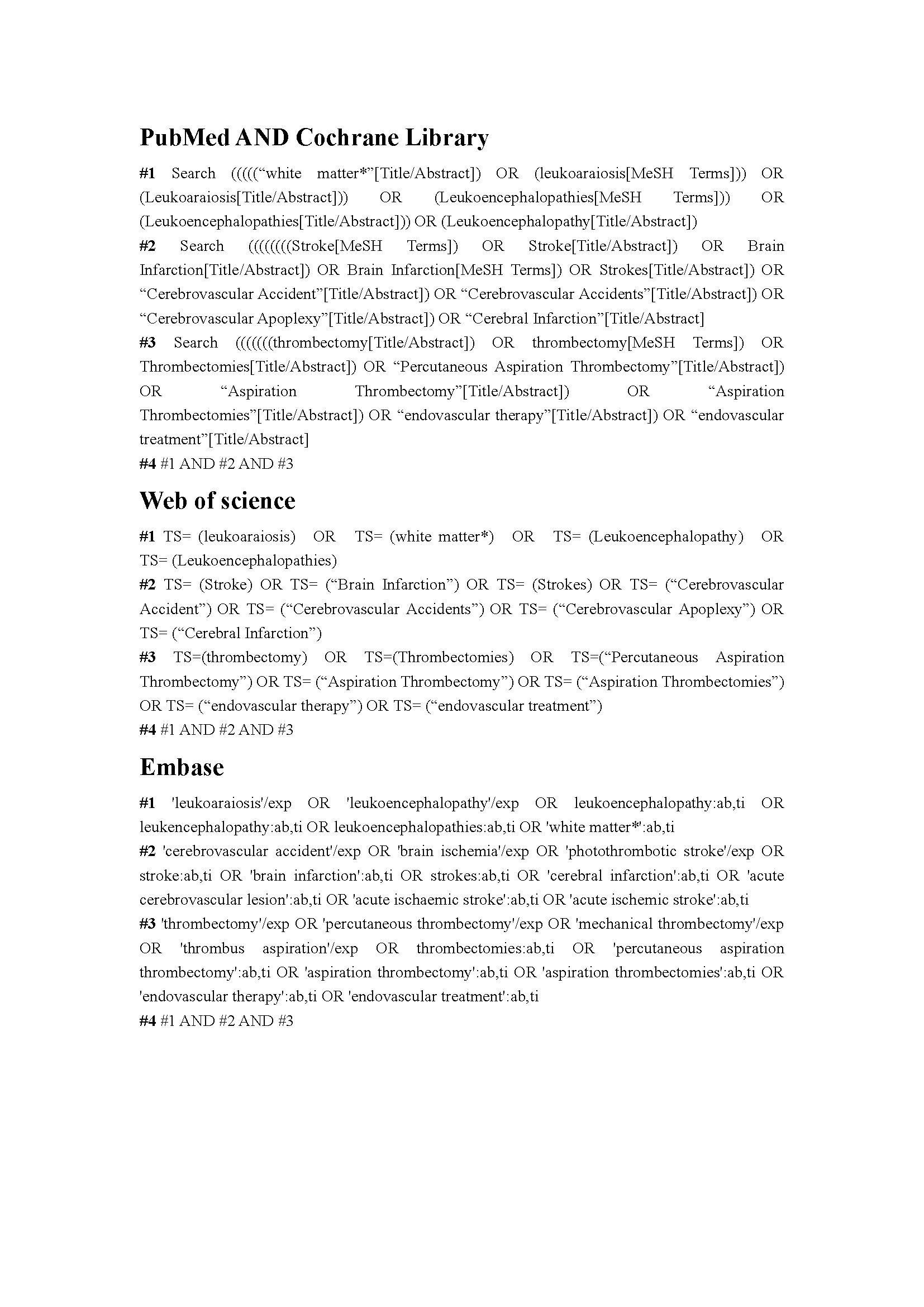

Supplement: Supplementary file 1 — Supplementary file1 (TIFF 382kb) [file 415_2020_10167_MOESM1_ESM.tif]

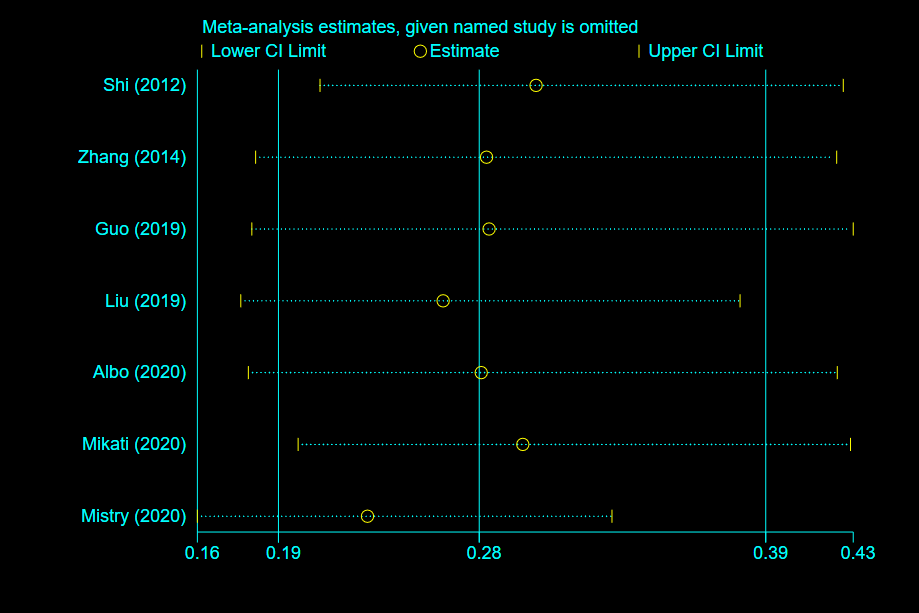

Supplement: Supplementary file 2 — Supplementary file2 (PNG 49kb) [file 415_2020_10167_MOESM2_ESM.png]

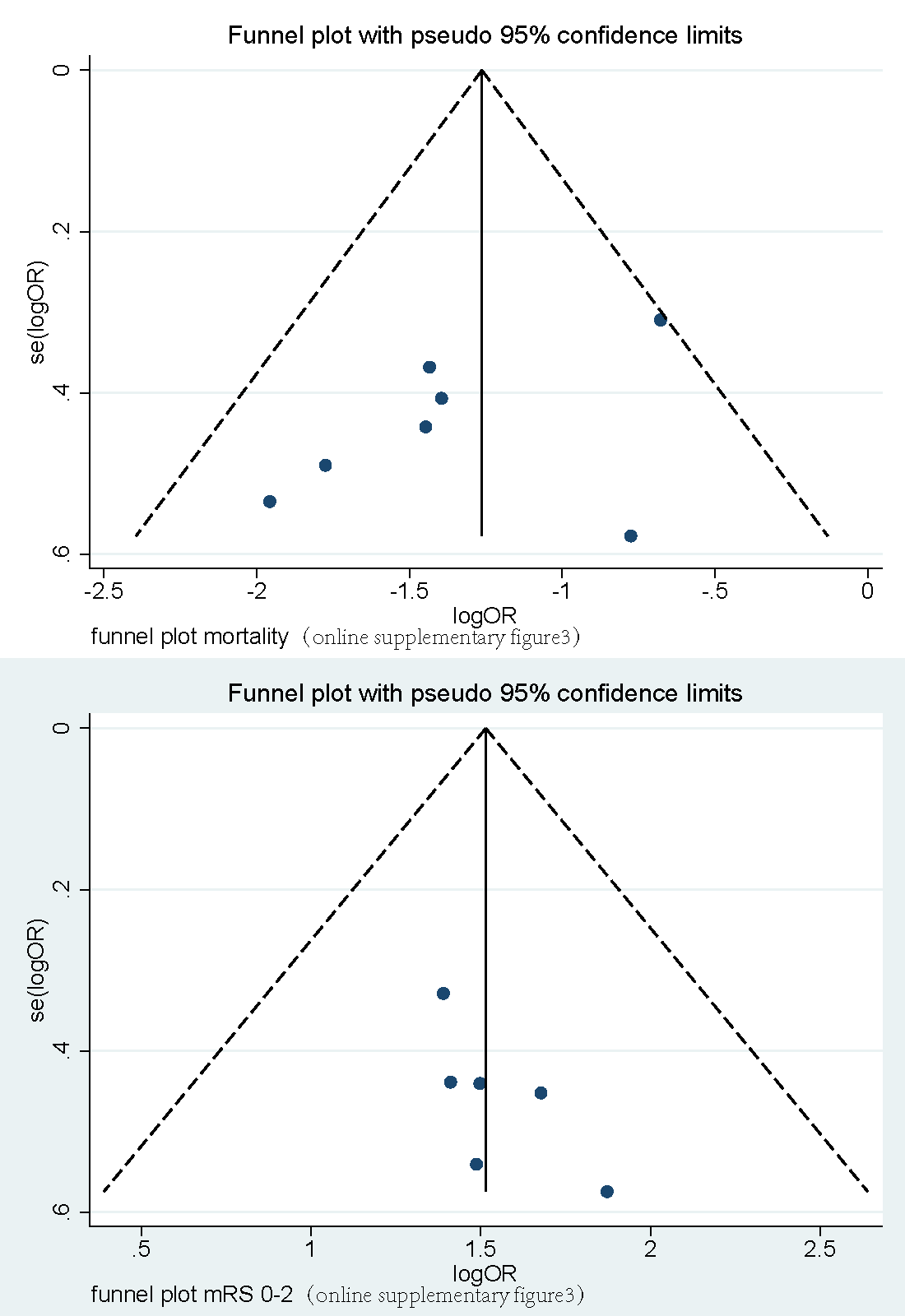

Supplement: Supplementary file 3 — Supplementary file3 (TIFF 5522kb) [file 415_2020_10167_MOESM3_ESM.tif]
